# Ten reasons to not ignore the third molar

**DOI:** 10.1590/2177-6709.26.1.e21ins1

**Published:** 2021-03-10

**Authors:** Alberto CONSOLARO, Omar HADAYA

**Affiliations:** 1 Universidade de São Paulo, Faculdade de Odontologia de Bauru (Bauru/SP, Brazil).; 2 Universidade de São Paulo, Faculdade de Odontologia de Ribeirão Preto (Ribeirão Preto/SP, Brazil).; 3 Digital Center Radiologia (Maringá/PR, Brazil).

**Keywords:** Third molars, Pericoronaritis, Paradental cyst, Tooth resorptions, Unerupted teeth, Dentigerous cyst

## Abstract

**Introduction:**

The third molars are forgotten because they are the last in the dental arch, they do not directly influence the smile and they appear only in adolescence, when they do.

**Objectives:**

1) to provide the clinician with a “checklist” to assess and diagnose changes to be screened in the third molar region in new patients; 2) to reveal the importance of not discharging the patient submitted to any dental treatment without first analyzing the third molars region clinically and on imaging examinations, since many diseases are associated to them.

**Result:**

A list of 10 situations that cover all diagnostic possibilities involving the third molars is presented.

**Conclusion:**

Adopting this protocol is a matter of habit, since the need is fundamental. The next professional assisting your patient may ask: *“Did he not request examinations for the third molars?”*.

## RESUMO

**Introdução:**

Os terceiros molares são esquecidos por serem os últimos dentes na arcada dentária, por não influenciarem diretamente no sorriso e por aparecerem apenas na adolescência - quando aparecem.

**Objetivos:**

1) Fornecer ao clínico um *checklist* de conferência e diagnóstico de alterações a serem checadas na região dos terceiros molares em novos pacientes; e 2) Destacar a importância de não dar alta ao paciente submetido a qualquer tratamento odontológico sem antes analisar, clínica e imagiologicamente, a região dos terceiros molares, pois muitas doenças estão a eles associadas.

**Resultado:**

Criou-se uma lista de 10 situações que englobam todas as possibilidades diagnósticas envolvendo os terceiros molares.

**Conclusão:**

Adotar esse protocolo é uma questão de hábito, pois a necessidade é imperiosa. O próximo profissional a atender o seu paciente vai perguntar: *“Ele não solicitou exames para os terceiros molares?”*.

## INTRODUCTION

It is not uncommon for us to examine only the tooth related to the main complaint of the patient, such as painful sensitivity, gingival recession and/or an esthetic change, or even an enamel fracture and/or a poorly fitting restoration.

It is also not uncommon to focus only on the expectation due to which the patient sought the professional, such as whitening, a facet, dental alignment or restoration.

Unfortunately, many patients finish their orthodontic treatments, even with orthognathic surgeries, with upper and/or lower third molars with anomalies of position and eruption, inducing resorptions in neighboring teeth, with pericoronitis and paradental cysts.

Ideally, after the first consultation, we should request a panoramic radiograph and periapical radiographs of the jaws. The surprise will always be present, as dental anomalies, periapical lesions, tooth resorptions, intraosseous lesions, calcific metamorphoses of the pulp, supernumerary teeth, unerupted teeth, etc.

With the images and an imaging report in hand, you will feel a complete professional in planning, and the patient will feel much reassured about the professional caring for his or her oral health. This routine will make you a happier professional!

In the third molar region, embryonic odontogenic tissues are exposed for many years to intrinsic and extrinsic environmental factors, as well as growth and lack of space, which combine and increase the possibility of problems related to anomalies of shape, position and eruption. This region should be checked for each new patient to prevent resorption, pericoronitis, paradental cysts, concrescences, cysts and odontogenic tumors. Thus, pain and mutilation are avoided, and the patient will always be close to the desired function and esthetics.

The aim of this work is to provide the clinician with a “checklist” or a protocol to assess and diagnose changes to be screened in new patients, regarding the third molar region. The other objective is to reveal the importance of not discharging the patient submitted to any dental treatment, including orthodontic treatment, before analyzing the third molars region clinically and by imaging examinations.

## CHECKLIST FOR THIRD MOLARS

### IN ALL DENTAL PATIENTS, INCLUDING ORTHODONTIC PATIENTS

**Figure 1:**
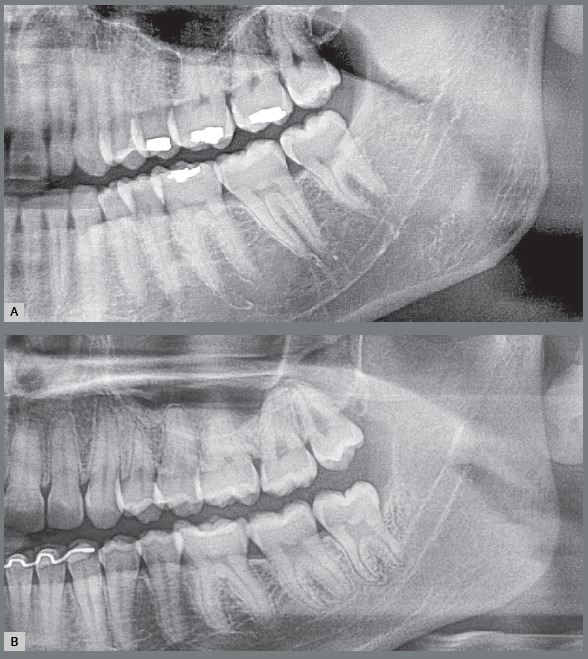
Third molars erupted in occlusion. Ideally, there should be gingiva distal to the third molar at the cervical level, such as in tooth #28. If there is lack of distal bone space, as in tooth #38, the gingiva is at the occlusal level, favoring the development of pericoronitis and a paradental cyst.

**Figure 2:**
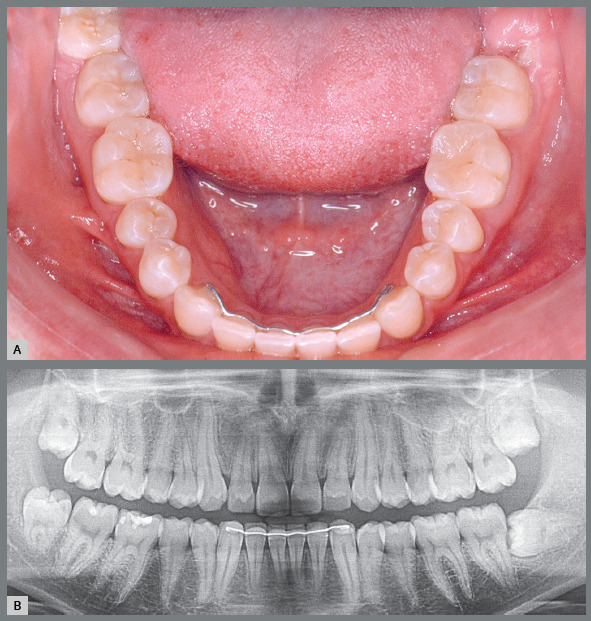
Lower and upper third molars with risk of pericoronitis, paradental cyst and external root resorption in the distal surface of second molars in a patient discharged from orthodontic treatment.

**Figure 3:**
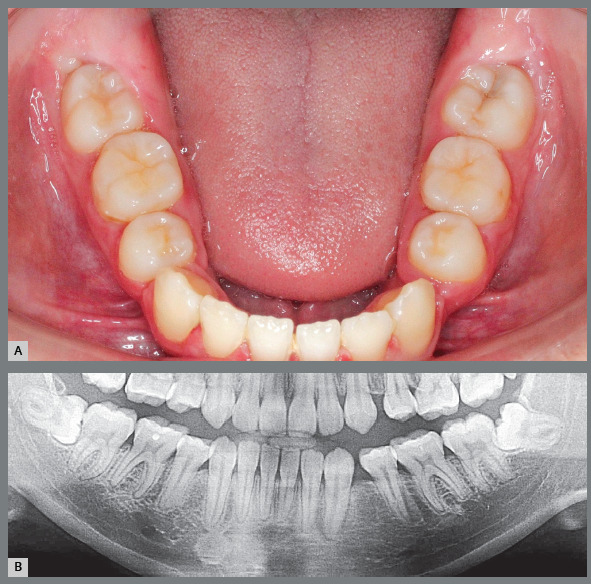
Small gingival cap or operculum in the lower third molars with aspect of normal mucosa revealing communication with the oral environment, increasing the risk of episodes of pericoronitis and development of paradental cyst. On the panoramic radiograph, the horizontal and mesially angulated position of the lower third molars reveals a risk of external root resorption in second molars.

**Figure 4:**
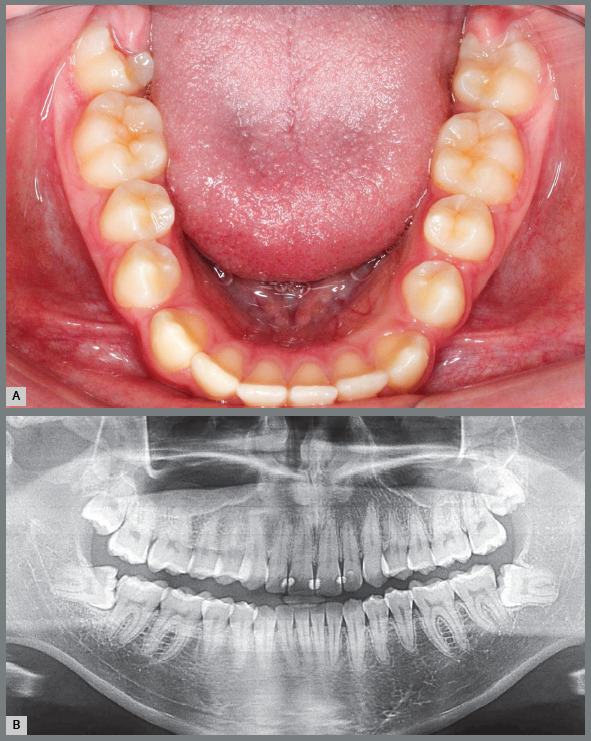
Hyperplastic gingival opercula or hyperplastic caps in the third molars create an interface that protects microbial biofilms, increasing the risks of pericoronaritis and a paradental cyst. On the panoramic radiograph, the horizontal and mesially angulated position of the lower third molars reveals a risk of external root resorption in the second molars.

**Figure 5:**
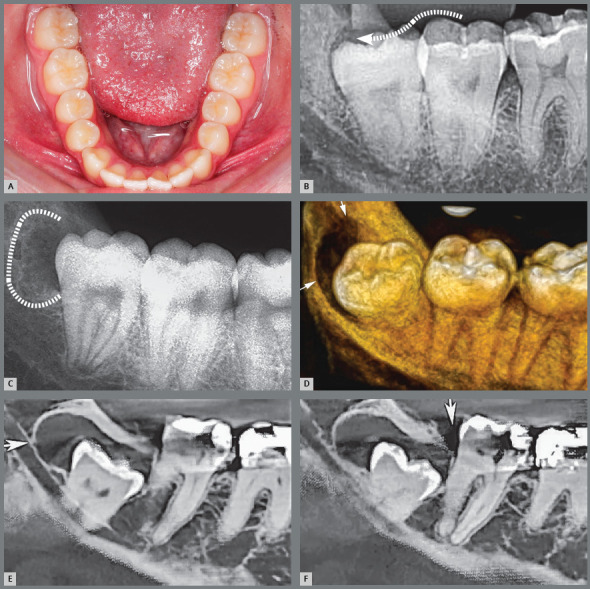
Bacteria and microbial biofilms infiltrate the partially erupted third molar follicle and communicates with the oral environment (dotted arrow, in **B**). Episodes of acute and chronic pericoronaritis develop, alternating with irregular distal bone resorption (dotted line, in **C**). Thus, the paradental cyst is formed (smaller arrows).

**Figure 6:**
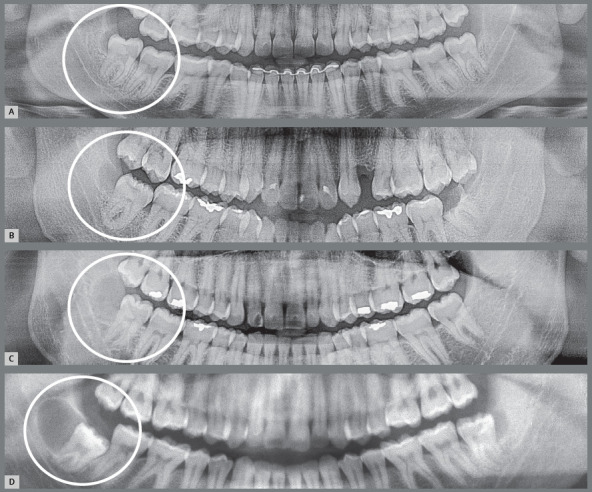
Paradental cysts from incipient development up to advanced occurrence on the distal surface of partially erupted lower third molars on the right side of panoramic radiographs. It is noteworthy that the patient in **A** was discharged from orthodontic treatment.

**Figure 7:**
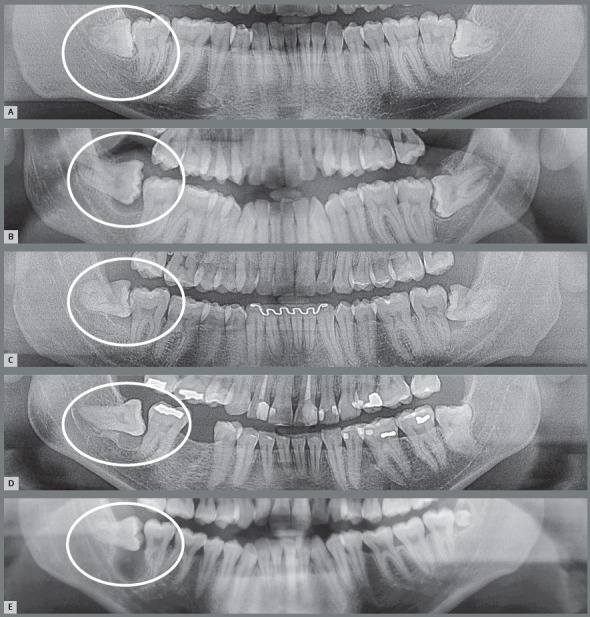
Paradental cysts from normal pericoronal space to the development on the mesial surface of the partially erupted lower third molars, mesially angulated and horizontalized on the right side of the panoramic radiographs. It is noteworthy that the patient in **C** was discharged from previous orthodontic treatment and was also affected on the left side.

**Figure 8:**
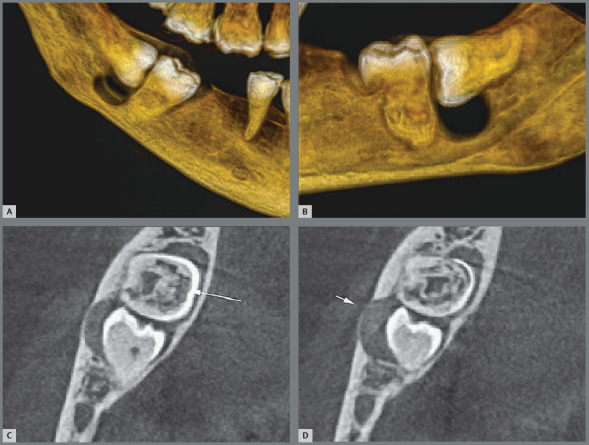
Mesial paradental cyst on the partially erupted lower third molar associated with partially erupted lower second molar with ankylosis and replacement resorption. Perforation of the buccal and lingual cortical bone is highlighted.

Ask yourself and your patient: where and how are the third molars. They can be:

1. Erupted and in normal occlusion with the antagonist, which is ideal and desired, yet unfortunately it does not always happen (Fig 1). If the antagonist is not present, its tendency is to extrude and cause periodontal and sensitivity problems.

2. Erupted and in normal occlusion with the antagonist, yet without distal bone space to form a healthy gingiva (Fig 1). The gingiva may be occupying the entire distal aspect of the crown with a long gingival sulcus, with difficult hygiene. In the site there may be gingivitis, periodontitis, pericoronitis and their consequences, including contribution to halitosis and constant perception of oral bleeding, altering the sense of taste.^1^ ^,^ ^2^

3. Erupted in normal occlusion, yet with a pericoronal cap over the occlusal surface coming from the distal part of the buccal/gingival mucosa (Figs 2, 3 and 4). This pericoronal cap can be identified as a gingival operculum or it may be also called a cap. A long interface is formed up to the distal cervical region of the tooth, accumulating microbial biofilms, food debris and predisposing the site to episodes of acute and chronic pericoronaritis alternately (Fig 5). At each episode, there is deepening and widening of this distal gingival interface and a paradental cyst is established in its distal part (Fig 6).^1^ ^,^ ^2^ ^,^ ^3^ ^,^ ^4^

4. Partially erupted and not impacted, despite the mesial angulation, with paradental cyst, yet still not impacting the second molar, which would prevent them from reaching the occlusal plane, greatly increasing the possibility of acute and chronic pericoronaritis alternately to give rise to a paradental cyst on its distal or mesial aspect towards the mandibular base (Figs 7 and 8).^1^ ^,^ ^2^ ^,^ ^3^ ^,^ ^4^ On the face opposite to that occupied by the cyst, the gingival tissues may be normal.

In this situation, the crown may be turned toward the buccal or lingual sides, yet in contact with the oral environment, its microbiota and food debris (Figs 5 and 6). The chance of alternate episodes of acute and chronic pericoronitis giving rise to paradental cyst increases considerably on its distal aspect in this situation.^2^

**Figure 9:**
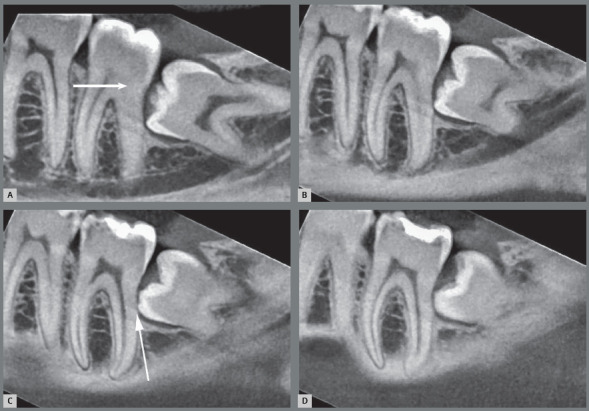
Incipient external inflammatory resorption on sagittal tomographic images. The partially erupted third molar with its pericoronal follicular tissues, combined to the force of the eruption pathway, can compress the vessels due to lack of oxygenation and eliminate the cementoblasts on the root of the associated lower second molars, thus initiating an external inflammatory resorption process (arrows).

**Figure 10:**
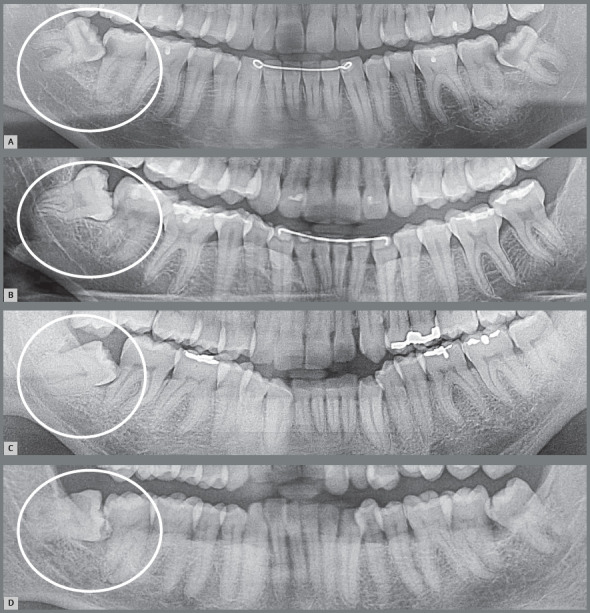
External inflammatory resorption on the distal surface of roots of the right lower second molars by direct action of the pericoronal follicular tissues of the lower third molars that compressed the vessels and led to death of cementoblasts. This resorptive process can be accelerated by the inflammation associated with the entry of bacteria via the distal gingival sulcus of the second molar and by partial exposure of the crown in the oral environment (arrow). Two patients (**A** and **B**) who were discharged from orthodontic treatment are highlighted.

**Figure 11:**
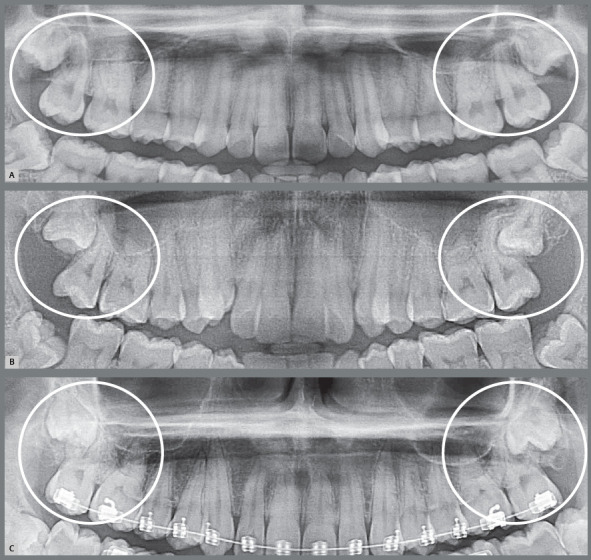
External inflammatory resorption on the distal surface of roots of the right and left upper second molars, by direct action of the pericoronal follicular tissues of the upper third molars. This resorptive process can be accelerated by the inflammation associated with the entry of bacteria through the distal gingival sulcus of the second molar and by partial exposure of the crown in the oral environment (arrow). The patient undergoing orthodontic treatment is highlighted in **C**.

**Figure 12:**
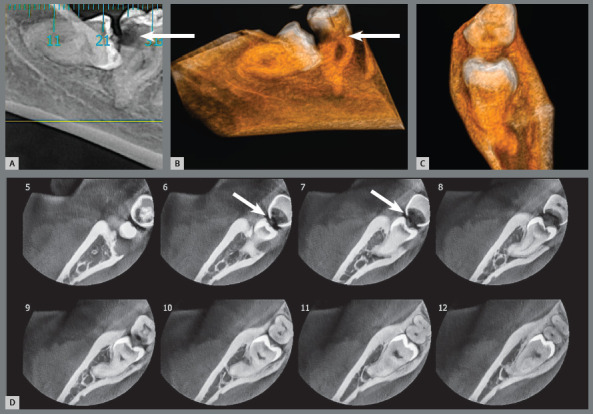
External cervical resorption in the second molar associated with exposure of the cementoenamel junction to follicular tissues of the partially erupted third molar (3D reconstruction in **B** and **C** and coronal sections in **D**).

5. Partially erupted and impacted on the second molars, with Inflammatory external root resorption due to the pericoronal follicular tissues in contact and interacting with the gingival and ligament tissues (Fig 9).^5^ ^,^ ^6^ There will be compression of vessels, eliminating the cementoblasts from the root surface and initiating an inflammatory external root resorption on the distal surface of lower (Fig 10) and upper second molars (Fig 11).

6. Partially erupted, mesially angulated and impacted on the second molars, with inflammatory external cervical resorption from the cementoenamel junction of these teeth (Fig 12). The pericoronal follicles and associated inflammation digest the extracellular connective tissue matrix that hid the dentin in exposed micro-windows. The exposed dentin tends to be resorbed because it stimulates the immune system, since it has six proteins recognized as foreign by our body.^5^ ^,^ ^6^

7. Unerupted, without impaction on neighboring teeth, which occurs due to lack of bone space in the alveolar process. They are asymptomatic and are not even noticed by the patient, but due to their position they end up impacting the second molars over time or remain indefinitely unerupted.

Without a minimum masticatory function, the periodontal ligament becomes markedly atrophic over time and the teeth may eventually become ankylosed and undergo replacement tooth resorption, disappearing completely.^5^ ^,^ ^8^ ^,^ ^9^

**Figure 13:**
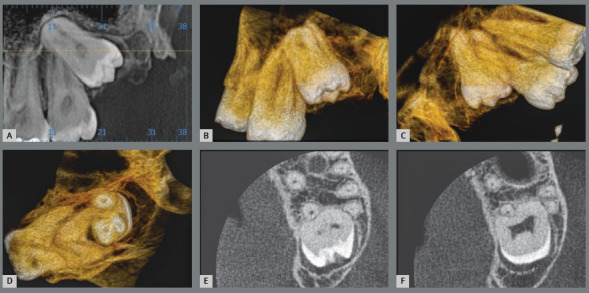
Concrescence of the upper third molar with the second molar on panoramic (**A**), axial (**E** and **F**) and 3D (**B**, **C** and **D**) tomography reconstructions, with buccal/palatal and axial views.

In some occasional cases, the lack of eruption and without impaction, especially in upper third molars, the excessive proximity to the periodontal ligaments, being one of them without minimum masticatory function, can lead to concrescence^10^ with the second molar (Fig 13).

**Figure 14:**
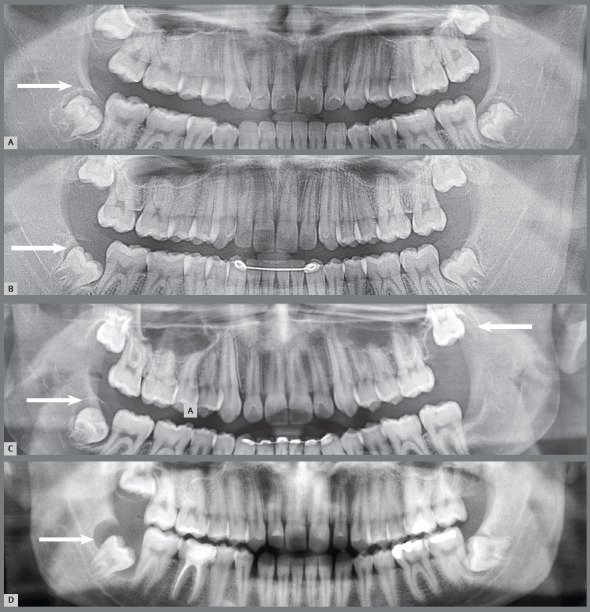
Dentigerous cysts: from normal pericoronal space to a gradual increase in thickness, causing the cystic cavity (arrows) by a process resulting from the eruptive process without microbial contamination, collecting liquid between the pericoronal follicular epithelium and enamel, without inflammatory process. In **B**, the patient who was discharged from orthodontic treatment is highlighted.

8. Unerupted without impaction on neighboring teeth, with dentigerous cyst. This situation is occasional and occurs due to accumulation of fluid between the reduced enamel epithelium and the dental enamel (Fig 14). Supposedly the dentigerous cyst occurs due to venous compression in the eruptive movement, yet this is still only a theory to explain its mechanism of formation. The edema would lead to liquid accumulation between both structures. The dentigerous cyst is typical of dental development, while the paradental cyst is inflammatory induced by previous acute and chronic pericoronaritis, i.e., it has another mechanism of formation.^3^ ^,^ ^4^ ^,^ ^6^

The pericoronal follicle has a thickness of up to 5.6 mm; above this, it should be considered a dentigerous cyst. Due to image distortion, this measure can make the case in a borderline situation. The greater the thickness of the follicular space, the more likely it is to be a dentigerous cyst. This situation is defined by the outflow of liquid between the follicle and enamel during transoperative surgical procedures. In these cases, it is also microscopically difficult to distinguish a pericoronal follicle from a dentigerous cyst.^1^ ^,^ ^2^ ^,^ ^3^ ^,^ ^4^ ^,^ ^6^

**Figure 15:**
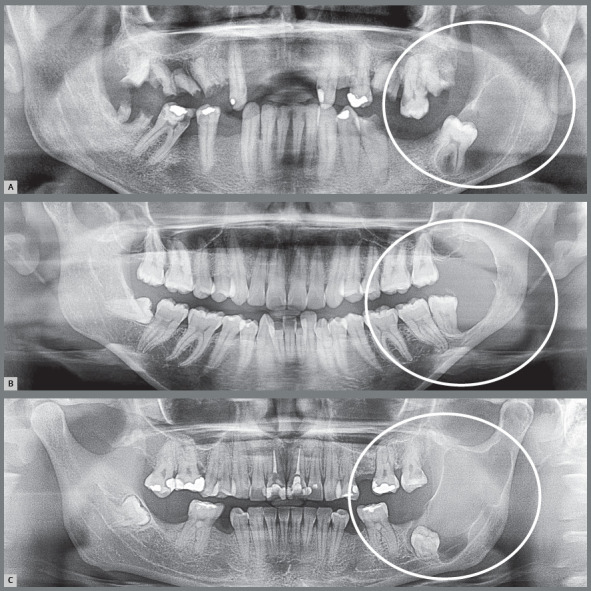
Odontogenic keratocysts that mimic the dentigerous cyst and even ameloblastoma, originated from dental lamina rests of the pericoronal follicle. The odontogenic keratocyst occurs is the posterior region of the mandible, especially associated with the third molars.

9. **Unerupted, without impaction on neighboring teeth, with odontogenic keratocyst.** Up to 30 years of age, the pericoronal follicle is rich in odontogenic epithelium islets derived from the dental lamina, which can give rise to odontogenic keratocyst. When developing in the pericoronal follicle, the odontogenic keratocyst will present the imaging and clinical aspect of dentigerous cyst for a long time (Fig 15). In most cases, the microscopic diagnosis of odontogenic keratocyst surprises the clinician and radiologist when receiving the histopathological report.^1^ ^,^ ^2^ ^,^ ^3^ ^,^ ^4^ ^,^ ^6^

10. Unerupted, without impaction on neighboring teeth, with ameloblastoma. The odontogenic epithelium islets may also eventually give rise to odontogenic tumors of the most varied types, including odontomas, ameloblastomas and other less common (Fig 15). This possibility is very small considering that there are many people with unerupted teeth, and the cases are occasional. It is so small that unerupted teeth may be followed with imaging examinations, in positions that would require major surgery on the mandibular ramus, orbit floor and mandibular base; or in cases of unerupted teeth in patients with systemic diseases as diabetes, autoimmune disorders, elderly individuals and other situations.

After 30 years of age, the odontogenic islets and epithelia undergo atrophy and then apoptosis, decreasing the possibility of odontogenic cysts and tumors starting after this stage of life^3^. Sometimes the possibility of an unerupted tooth giving rise to an odontogenic tumor such as ameloblastoma is greatly reinforced because of an inverted analysis of the problem: half of cases of odontogenic tumor are associated with unerupted teeth! Either because they give rise to them or because they prevent their eruption, but even so the number of cases in the population is very small, considering the number of teeth and the number of people, so that it is not possible to speak in percentage, since it would be extremely small in number.

## FINAL CONSIDERATIONS

A dental patient should not be discharged without being sure that he or she does not have a disease in the jaws. For this reason, in any planning, even for a simple restoration, it is important to have panoramic and periapical radiographs of the jaws in hands.

Unfortunately, many patients finish their treatments and are discharged by the professional, yet the third molars present anomalies of position or eruption, inducing resorptions in neighboring teeth, with pericoronitis and paradental cysts, besides neoplasms.

Working on dental crowns requires knowledge on how the periodontium and underlying bone are, always thinking about the patient as a whole also over time. The diseases, even the most serious, start small and with asymptomatic signs. The early diagnosis can prevent further damage and functional and structural mutilation.
